# Urinary Nerve Growth Factor Could Be a Biomarker for Interstitial Cystitis/Painful Bladder Syndrome: A Meta-Analysis

**DOI:** 10.1371/journal.pone.0106321

**Published:** 2014-09-02

**Authors:** Hong-Chen Qu, Wei Zhang, Shi Yan, Yi-Li Liu, Ping Wang

**Affiliations:** 1 Department of Urological Surgery, The Fourth Affiliated Hospital of China Medical University, Shenyang, Liaoning, P. R. China; 2 Department of Orthodontics, The Dental Affiliated Hospital of China Medical University, Shenyang, Liaoning, P. R. China; 3 Department of Urological Surgery, The Third Hospital of Shenyang, Shenyang, Liaoning, P. R. China; Oklahoma University Health Sciences Center, United States of America

## Abstract

To examine whether urinary nerve growth factor (NGF) could serve as a biomarker for interstitial cystitis/painful bladder syndrome (IC/PBS), we conducted a comprehensive meta-analysis of 9 studies. Among the studies considered, patients with IC/PBS had higher urinary NGF and NGF/Cr levels compared to those of healthy people (SMD = 1.94, 95%CI = 0.79–3.08, P = 0.0009 and SMD = 1.79, 95%CI = 0.65–2.93, P = 0.002, respectively). In addition, there was a significant difference between patients with IC/PBS and patients with overactive bladder (OAB) symptoms with respect to the urinary NGF and NGF/Cr levels (SMD = −0.62, 95%CI = −1.00–−0.24, P = 0.001 and SMD = −0.70, 95%CI = −1.01–−0.39, P<0.0001, respectively). Furthermore, patients had a significantly lower urinary NGF level after successful treatment (SMD = 1.74, 95%CI = 0.32–3.17, P = 0.02). In conclusion, urinary NGF could be a useful biomarker for the diagnosis of OAB, a urinary biomarker for the differential diagnosis of IC/PBS and OAB (when a critical urinary NGF or NGF/Cr level is needed), and a predictive biomarker to help guide treatment.

## Introduction

Interstitial cystitis/painful bladder syndrome (IC/PBS) is a chronic, painful inflammatory disorder of the urinary bladder that is characterized by pelvic pain and irritative voiding symptoms [1]. Approximately 2%–11% of adult men and women in the United States have IC/PBS-like symptoms [2], [3]. Patients with IC/PBS present with bladder pain and frequent voiding, whereas patients with overactive bladder (OAB) symptoms present with urgency and sometimes urgency incontinence. IC/PBS or OAB is usually diagnosed according to subjective symptoms and cystoscopic hydrodistension following the exclusion of other bladder disorders [4]. However, OAB symptoms always overlap with symptoms of IC/PBS, and not all patients with IC/PBS present a cystoscopic hydrodistension finding [5]. Therefore, to avoid the expense and the risk of expensive and invasive procedures (such as cystoscopy and tissue biopsy), it is necessary to establish a noninvasive diagnostic tool that can be used to diagnose IC/PBS and differentially diagnose IC/PBS and OAB.

The term “biomarker” generally indicates anything that can be used to describe a particular disease state, including its presence (diagnostic biomarker), severity, and/or its response to a specific treatment (predictive biomarker). Biomarkers can be specific cells, enzymes, hormones, genes, or gene products and can be detected and measured in various parts of the body, such as the blood, urine, or tissue [6]. Nerve growth factor (NGF) is a signaling protein produced by the bladder smooth muscle and urothelium [7]. Previous studies have shown a direct link between painful inflammatory conditions in the lower urinary tract and increased NGF levels in the bladder tissue and urine [7]–[11]. In a rat model of IC [11], the NGF level in inflamed bladder tissue was higher than that in controls (bladder inflammation caused a 50% increase in NGF), and the increase in NGF was most pronounced after mechanical inflammation. Steers WD [8] and Tuttle JB [9] discovered that NGF plays a significant role in regulating the growth of the bladder. Furthermore, Steers WD et al also revealed that the ability of NGF to trigger interstitial cystitis might rely on altering the properties of sodium or potassium channels in bladder afferent fibers [7]. Based on these previous studies, there has been a great interest in studying NGF as a potential biomarker for patients with IC/PBS. Therefore, we performed a meta-analysis of the most recent and relevant articles to evaluate the potential for using NGF as a biomarker for IC/PBS. These evidence-based findings could provide urologists with evidence supporting the role of urinary NGF in the diagnosis of IC/PBS.

## Material and Methods

### 1 Literature search

We performed an electronic search of the PubMed, Cochrane Library, Embase, Web of Science, Springer Link, and CBM databases to identify relevant studies reported as recently as April 4, 2014. The search terms included [‘interstitial cystitis/painful bladder syndrome’ or ‘IC/PBS’ (MeSH)] and [‘nerve growth factor’ or ‘NGF’ (MeSH)]. Other relevant studies cited in the reference lists of the selected papers were also evaluated to identify additional eligible studies.

### 2 Inclusion and exclusion criteria

To be eligible, the included studies were required to meet the following criteria: i) the study was a randomized controlled trial (RCT) or a case-control study; ii) the study focused on the association between urinary NGF levels and IC/PBS; iii) all patients were diagnosed with clinical IC/PBS (suprapubic pain with a full bladder that was relieved after voiding associated with severe frequency and nocturia); iv) the original data for the dichotomous and continuous variables was provided or could be calculated from the data source. Additionally, the eligible studies were limited to those that were only in English. Studies were excluded if they reported incomplete, useless, or overlapping data; if they used median and interquartile ranges to describe the data; or if they were published only as abstracts, letters, reviews, or reports from academic meetings.

### 3 Data extraction

Using a standardized form, the data from the published studies were independently extracted by two reviewers (Hong-Chen Qu and Wei Zhang) to populate the necessary information. The following information was extracted from each of the articles: first author, year of publication, study design, number of cases and controls, mean age, sample, clinical symptoms, treatment, and urinary NGF (or NGF/Cr) levels. In cases of conflicting evaluations, an agreement was reached following a discussion with a third reviewer (Ping Wang).

### 4 Quality assessment of the included studies

Two reviewers (Hong-Chen Qu and Wei Zhang) independently assessed the quality of the publications according to a modified STROBE quality score system [12], [13]. In total, 22 assessment items related to the quality appraisal were used in this meta-analysis, and the scores ranged from 0 to 44. Scores of 0–17.5, 17.5–35, and 35–44 were defined as poor, fair, and good, respectively. Disagreements were resolved by discussion.

### 5 Statistical analysis

To ensure the reliability and the accuracy of the results, 2 independent authors not involved in data collection (Shi Yan and Yi-Li Liu) independently populated the data in the statistical software programs to confirm the results. The standard mean difference (SMD) and 95%CI were calculated using Review Manager Version 5.1.6 software (provided by the Cochrane Collaboration). Between-study heterogeneities were estimated using Cochran's Q-statistic [14], [15] (P≤0.05 was considered a manifestation of significant heterogeneity). We also quantified the effect of heterogeneity using the I^2^ test, which ranged from 0 to 100% and represented the proportion of inter-study variability that could be contributed to heterogeneity rather than chance. The data were pooled using both fixed-effects and random-effects models. When a significant Q-test (P≤0.05) or I^2^ test >50% indicated the presence of heterogeneity among the studies, the random effects model was utilized because the random effects model provides a more conservative estimate of the inter-study variance. Otherwise, the fixed-effects model was used. Funnel plots were used to identify potential publication bias. In the funnel plot, an asymmetrical plot suggests a possible publication bias. Egger's linear regression test, which measures funnel plot asymmetry according to the natural logarithm scale of the OR, was used to evaluate the publication bias [16]. When the P value was <0.05, publication bias was considered significant. All P values were two-sided. Sensitivity analysis was carried out according to the quality of the included studies.

## Results

### 1 Characteristics of the included studies

**Figure 1:**
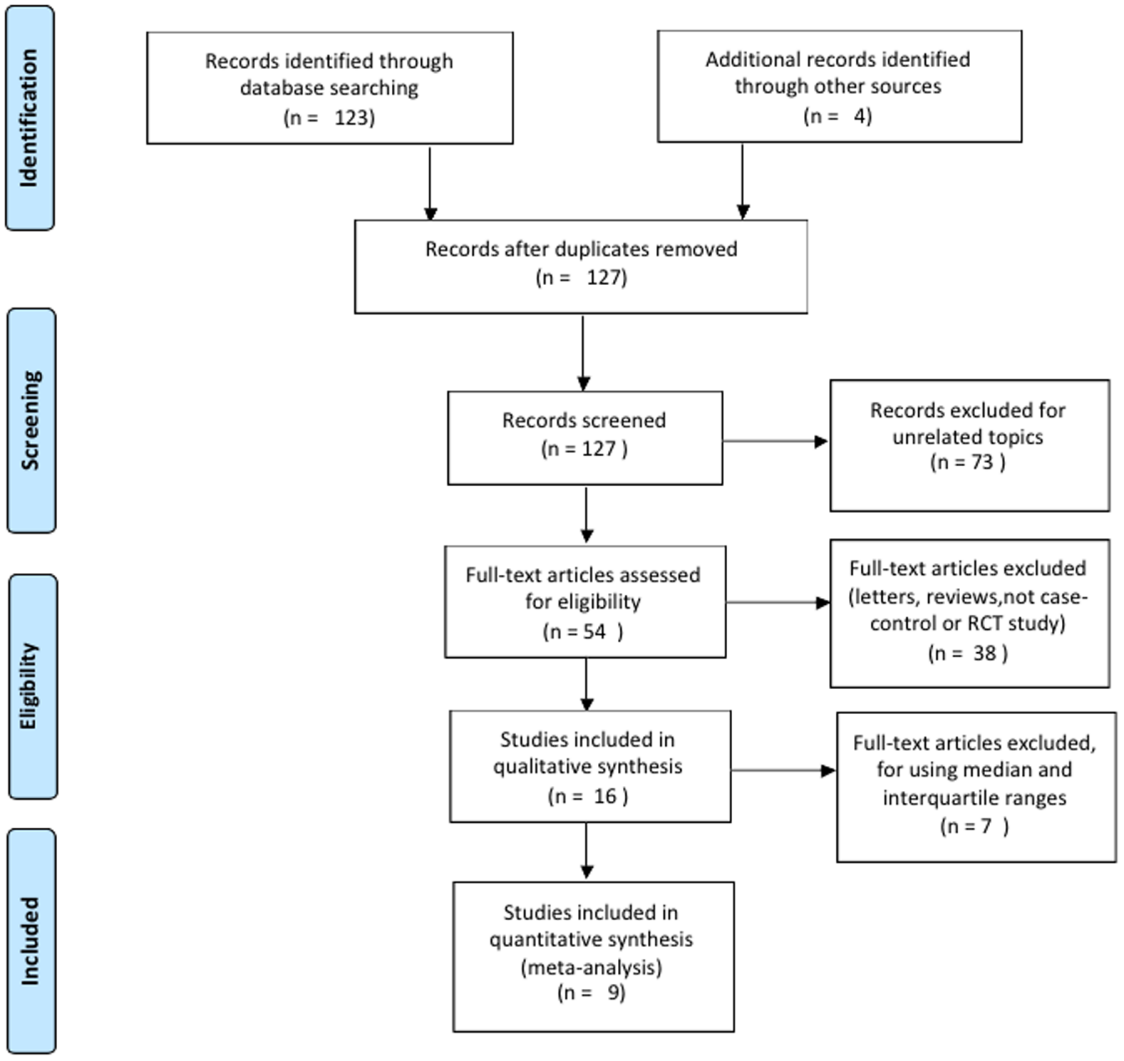
The flow chart of study selection. In this meta-analysis, 9 studies were selected for qualitative analysis. Among these 9 studies, 259 IC/PBS patients were included in our meta-analysis.

A total of 9 studies [17]–[25] met the inclusion criteria and were included in the meta-analysis. The flow chart of study selection is shown in Figure 1. The 9 studies included 259 patients with IC/PBS, 208 controls, and 67 patients with OAB. Overall, 102 patients received successful treatments. The publication years of the involved studies ranged from 2009 to 2014. All patients fulfilled the diagnostic criteria of clinical IC/PBS (suprapubic pain with a full bladder that was relieved after voiding associated with severe frequency and nocturia). The control patients were healthy individuals. The quality scores of the included studies were all more than 17.5 (fair or good). The characteristics and methodological quality of the included studies are summarized in Table 1.

**Table 1:** The characteristics and methodological quality of the included studies.

Author	Year	Number	Method	Quality score
Hsin-Tzu Liu	2009	IC/PBS (n = 58)	NGF	22
		Control (n = 28)	NGF/Cr	
		Pre-treatment (n = 43)		
		Post-treatment (n = 43)		
Hsin-Tzu Liu	2010	IC/PBS (n = 40)	NGF/Cr	23
		OAB (n = 23)		
		Control (n = 27)		
Rui Pinto	2010	Pre-treatment (n = 26)	NGF	22
		Post-treatment (n = 26)		
Hann-Chorng Kuo	2010	IC/PBS (n = 30)	NGF	26
		OAB (n = 22)	NGF/Cr	
		Control (n = 27)		
Shiu-Dong Chung	2011	IC/PBS (n = 48)	NGF	23
		OAB (n = 22)	NGF/Cr	
		Control (n = 33)		
Hsin-Tzu Liu	2012	IC/PBS (n = 30)	NGF	25
		Control (n = 28)	NGF/Cr	
Pradeep Tyagi	2012	IC/PBS (n = 10)	NGF	28
		Control (n = 10)		
Anthony T. Corcoran	2013	IC/PBS (n = 10)	NGF	24
		Control (n = 10)		
Yuan-Hong Jiang	2014	IC/PBS (n = 33)	NGF	28
		Control (n = 45)	NGF/Cr	
		Pre-treatment (n = 33)		
		Post-treatment (n = 33)		

IC/PBS: interstitial cystitis/painful bladder syndrome.

NGF: urinary nerve growth factor level.

NGF/Cr: urinary NGF level normalized to the urine Cr level.

OAB: overactive bladder.

### 2 Difference in urinary NGF and NGF/Cr (urinary NGF level normalized to the urine Cr level) levels in IC/PBS patients and healthy controls

**Figure 2:**
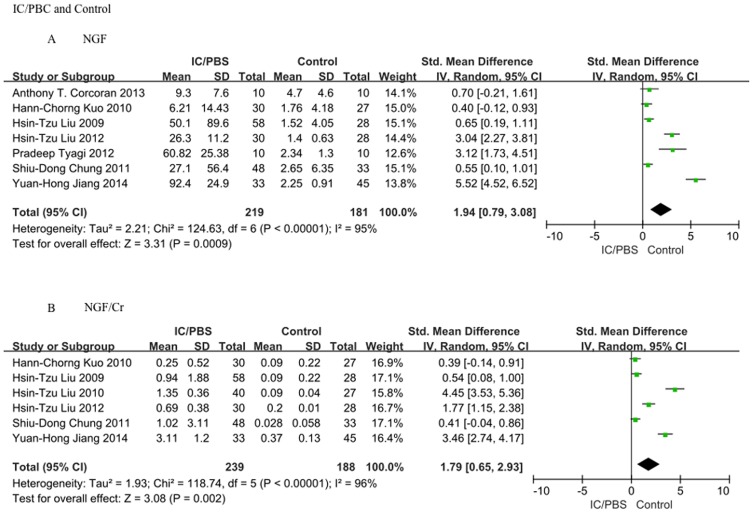
The association between the urinary NGF or NGF/Cr level and IC/PBS. A: NGF level. Interstitial cystitis/painful bladder syndrome (IC/PBS) patients presented a higher urinary NGF level compared to that of controls (SMD = 1.94, 95%CI = 0.79–3.08, P = 0.0009); B: NGF/Cr level. IC/PBS patients presented a higher urinary NGF/Cr level compared to that of controls (SMD = 1.79, 95%CI = 0.65–2.93, P = 0.002).

A summary of the meta-analysis findings regarding the difference in urinary NGF and NGF/Cr levels between IC/PBS patients and healthy controls is provided in Figure 2. Compared to controls, IC/PBS patients presented higher urinary NGF and NGF/Cr levels (SMD = 1.94, 95%CI = 0.79–3.08, P = 0.0009 and SMD = 1.79, 95%CI = 0.65–2.93, P = 0.002).

### 3 Difference in the urinary NGF and NGF/Cr levels of IC/PBS patients and patients with OAB symptom

**Figure 3:**
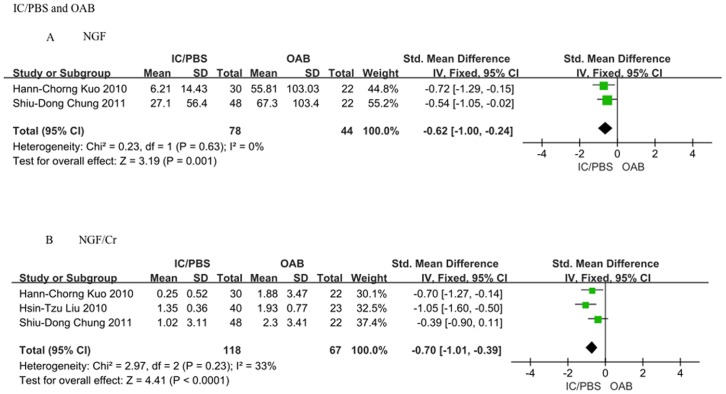
Differences in the urinary NGF and NGF/Cr levels between patients with IC/PBS and patients with OAB. A: NGF level. IC/PBS patients presented a lower urinary NGF level compared to that of overactive bladder (OAB) patients (SMD = −0.62, 95%CI = −1.00–−0.24, P = 0.001); B: NGF/Cr level. IC/PBS patients presented a lower urinary NGF/Cr level compared to that of OAB patients (SMD = −0.70, 95%CI = −1.01–−0.39, P<0.0001).

There was a significant difference between IC/PBS patients and patients with OAB symptoms with respect to the urinary NGF and NGF/Cr levels (Figure 3). The meta-analysis revealed that IC/PBS patients presented lower urinary NGF and NGF/Cr levels relative to patients with OAB symptoms (SMD = −0.62, 95%CI = −1.00–−0.24, P = 0.001 and SMD = −0.70, 95%CI = −1.01–−0.39, P<0.0001, respectively).

### 4 Difference in the urinary NGF levels of IC/PBS patients before and after successful treatment

**Figure 4:**
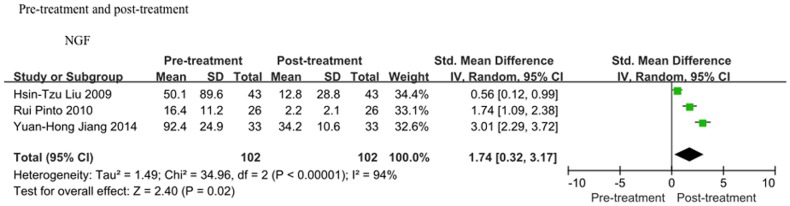
Differences in the urinary NGF levels of patients with IC/PBS before and after successful treatment. After successful treatment, IC/PBS patients presented a lower urinary NGF level (SMD = 1.74, 95%CI = 0.32–3.17, P = 0.02).

The urinary NGF levels of IC/PBS patients before and after successful treatment are shown in Figure 4. The meta-analysis revealed that the urinary NGF level was significantly reduced after successful treatment. The average decrease was 1.74 (SMD = 1.74, 95%CI = 0.32–3.17, P = 0.02).

### 5 Sensitivity analysis

**Figure 5:**
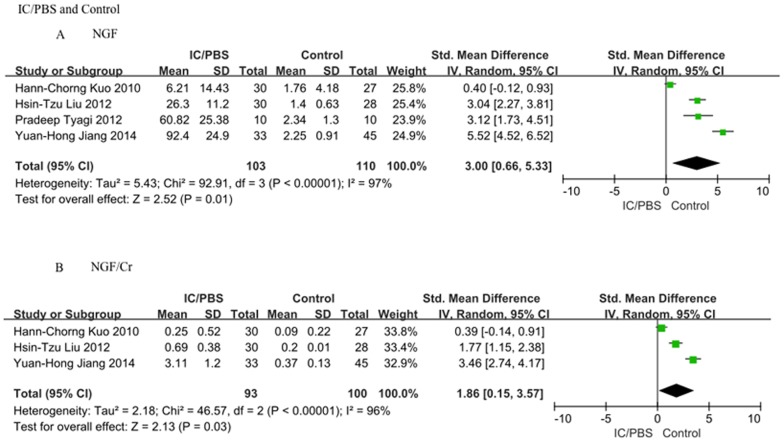
Sensitivity analysis. We excluded relatively low-quality studies (score<25) from the sensitivity analysis. A: NGF level. IC/PBS patients presented a higher urinary NGF level compared to that of controls (SMD = 3.00, 95%CI = 0.66–5.33, P = 0.01); B: NGF/Cr level. IC/PBS patients presented a higher urinary NGF/Cr level compared to that of controls (SMD = 1.86, 95%CI = 0.15–3.57, P = 0.03).

A sensitivity analysis was carried out by the quality of the included studies. We excluded relatively low-quality studies (score<25) and chose 4 high-quality studies (score≥25) to carry out the sensitivity analysis. Figure 5 shows that IC/PBS patients continued to present higher urinary NGF and NGF/Cr levels than those of controls (SMD = 3.00, 95%CI = 0.66–5.33, P = 0.01 and SMD = 1.86, 95%CI = 0.15–3.57, P = 0.03, respectively). The sensitivity analysis result was consistent with the previous result and statistically suggests that urinary NGF or NGF/Cr could be a credible and useful biomarker for the diagnosis of IC/PBS.

### 6 Publication bias

**Figure 6:**
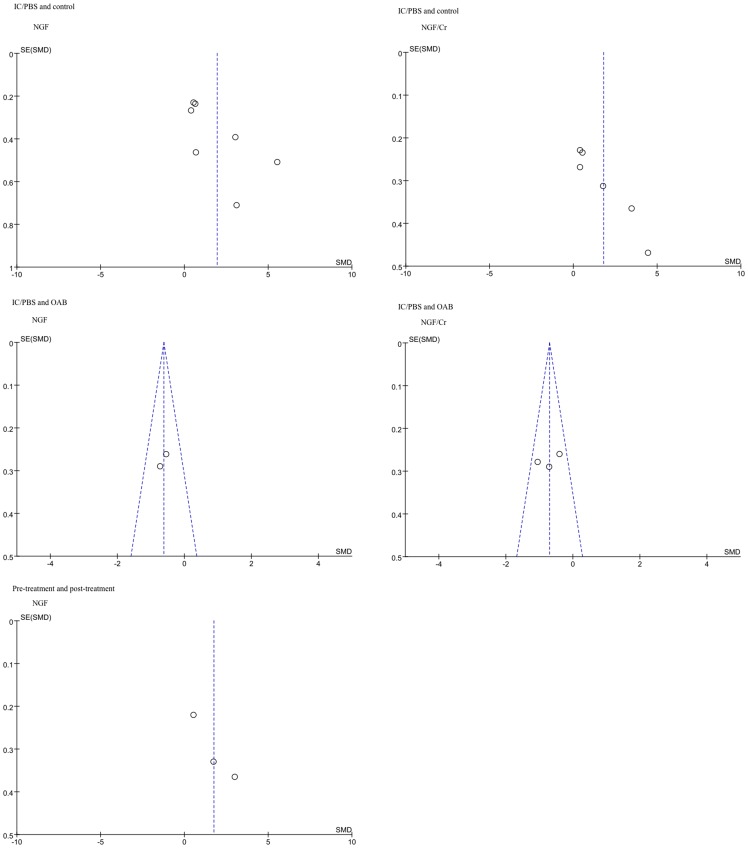
Graphical funnel plots of the included studies. These symmetrical plots indicate the absence of publication bias in the present meta-analysis.

The publication bias of the literature was assessed using a Begger's funnel plot and Egger's linear regression test. All of the graphical funnel plots of the included studies were symmetrical (Figures 6). Egger's test also indicated that there were no publication biases.

## Discussion

IC/PBS remains a debilitating bladder dysfunction of unknown etiology. A diagnosis of IC/PBS is made based on the characteristic symptoms of bladder pain and irritative voiding symptoms, including urgency [1]. Although the etiology of IC/PBS is not fully understood, chronic inflammation (whether triggered by a urinary tract infection or otherwise) is thought to be a potential mechanism of pathogenesis [26]–[28]. Ogawa et al found that chemokines, which play a pivotal role in the immune response and lead to inflammation, were over-expressed in the bladder urothelium of patients with interstitial cystitis [28]. Bladder inflammation in patients with IC/PBS leads to abnormalities or activation of the afferent sensory system in the urinary bladder or CNS sensitization, which can cause chronic pain symptoms, urgency and frequency in IC/PBS patients [7], [11], [29]. DuPont [11] evaluated chemical (2.5% formalin), immune (lipopolysaccharide 2×10^4^ cfu/ml) and mechanical (chromic catgut) inflammation at various times compared to control bladders. The study found that inflammation induces neuroplasticity of the sensory and motor neurons innervating the bladder, leading to long-term symptoms and pain. In addition, Amaya revealed that TRPV1 contributes to the development of inflammatory thermal hyperalgesia in IC/PBS patients [30]. Following a prior report that inflammation increases NGF expression, NGF has attracted considerable attention as a key player in the link between inflammation and IC/PBS.

NGF is a signaling protein expressed widely in various cells, including urothelial cells, smooth muscle cells and mast cells; it has been shown to stimulate mast cells to degranulate and proliferate [7]. NGF affects bladder afferent fibers and is responsible for the growth and maintenance of sensory neurons [31] and the function of adult visceral sensory and motor neurons [32]. Previous studies have reported an increased expression of NGF in bladder biopsies from women with IC/PBS [33], [34]. Lowe discovered that the levels of NGF were higher in samples obtained from patients with painful bladder conditions than from controls [33]. In addition, Liu [34] found that the NGF mRNA level was significantly higher in IC/PBS patients than in controls (0.65 +/− 0.33 versus 0.42 +/− 0.25, respectively, P = 0.046). Consequently, NGF has attracted considerable attention as a key player in the pathogenesis of IC/PBS. A basic science study reported that inflammation induces neuroplasticity, which leads to increased NGF levels in the bladder and generates IC/PBS [11]. In rats, intravesical bladder instillation with NGF induced bladder hyperactivity [35]. Both clinical and experimental data indicate that increased levels of NGF are found in the bladder tissue and urine of patients with IC/PBS [36].

We included 9 independent studies in the present meta-analysis to examine the association of urinary NGF levels in IC/PBS patients. Overall, our analysis indicated that patients with IC/PBS had higher urinary NGF and NGF/Cr levels than healthy controls (SMD = 1.94, 95%CI = 0.79–3.08, P = 0.0009 and SMD = 1.79, 95%CI = 0.65–2.93, P = 0.002, respectively). This result suggests that urinary NGF could be a potential biomarker for the diagnosis of IC/PBS. Furthermore, we identified that IC/PBS patients had significantly lower urinary NGF and NGF/Cr levels relative to those of OAB patients (SMD = −0.62, 95%CI = −1.00–−0.24, P = 0.001 and SMD = −0.70, 95%CI = −1.01–−0.39, P<0.0001, respectively). The symptoms of IC/PBS overlap with those of OAB, except that bladder pain typically presents in IC/PBS and urgency or urge incontinence presents in patients with OAB. As a result, IC/PBS can be diagnosed and treated as OAB. To overcome this problem, a biomarker is needed to differentiate between the two diseases. Rachaneni et al [37] discussed whether urinary NGF could serve as a biomarker of detrusor overactivity (DO) in patients with OAB. DO, which is defined as detrusor contractions during urodynamics, is responsible for symptoms in most OAB patients. The results of Rachaneni's systematic review [37] suggest an increasing trend of NGF in DO patients compared to controls. Our meta-analysis also indicated that urinary NGF could be used to make a differential diagnosis of IC/PBS and OAB. However, we were unable to calculate an accurate cut-off due to limited data. Therefore, well-designed randomized, controlled trials are still needed to identify the critical urinary NGF and NGF/Cr levels that can be used to precisely differentiate between IC/PBS and OAB. In addition, we found that urinary NGF levels decreased in response to effective treatment (SMD = 1.74, 95%CI = 0.32–3.17, P = 0.02). This result indicates that urinary NGF levels could be used to assess the therapeutic effects of OAB treatment. However, our meta-analysis has some limitations. First, the treatment schemes varied among the included studies (repeated hydrodistension, oral pentosan polysulfate, intravesical hyaluronic acid instillation, or intravesical botulinum toxin A injection). Furthermore, there were variations in the length of the follow-up period (ranging from 4 to 12 weeks after therapy). Both of these differences could have influenced our results. Therefore, results from a standard follow-up scheme need to be further investigated to confirm our findings.

Similar to other meta-analyses, this study has many limitations. First, the number of relevant research articles was small, and some studies included in this analysis evaluated a small number of patients. In addition, some of the relevant studies could not be included in our analysis because of incomplete raw data. Third, we were unable to address the sources of heterogeneity among all of the studies. Fourth, although the cases and controls of each study were well defined using similar inclusion criteria, certain potential factors were not considered, which might have influenced our results (for example, age, ethnicity, and timing of urine collection). Fifth, a meta-analysis is a retrospective study that is subject to certain methodological limitations. Most importantly, our meta-analysis was based on unadjusted SMD estimates because not all of the published studies reported adjusted SMDs or SMDs adjusted using the same potential confounders. Given these results, additional investigations into these areas are needed, and our conclusions should be interpreted cautiously.

## Conclusion

In conclusion, this meta-analysis of 9 studies demonstrates that the urinary NGF or NGF/Cr level could serve as a useful biomarker for the diagnosis of IC/PBS, a urinary biomarker for the differential diagnosis of IC/PBS and OAB and a predictive biomarker for specific treatments. Because of the limited number of published studies in this field, the current available evidence remains limited. Therefore, we emphasize the necessity of conducting a large, multicenter, well-designed, randomized, controlled trial with a long-term follow-up to confirm our findings.

## Supporting Information

Checklist S1 **PRISMA Checklist.**
(DOC)

## References

[1] Hanno PM, Landis JR, Matthews-Cook Y, Kusek J, Nyberg L Jr (1999) The diagnosis of interstitial cystitis revisited: lessons learned from the National Institutes of Health Interstitial Cystitis Database study. J Urol 161: 553–557. 9915447 10.1016/s0022-5347(01)61948-7

[2] Stewart WF, Van Rooyen JB, Cundiff GW, Abrams P, Herzog AR, et al (2003) Prevalence and burden of overactive bladder in the United States. World J Urol 20: 327–336. 12811491 10.1007/s00345-002-0301-4

[3] Clemens JQ, Meenan RT, O'Keeffe Rosetti MC, Brown SO, Gao SY, et al (2005) Prevalence of interstitial cystitis symptoms in a managed care population. J Urol 174: 576–580. 16006901 10.1097/01.ju.0000165170.43617.be

[4] Hanno PM, Sant GR (2001) Clinical highlights of the National Institute of Diabetes and Digestive and Kidney Diseases/Interstitial Cystitis Association scientific conference on interstitial cystitis. Urology 57: 2–6. 11378041 10.1016/s0090-4295(01)01112-8

[5] Messing EM (1987) The diagnosis of interstitial cystitis. Urol 2: 4–7. 3564233

[6] Bhide AA, Cartwright R, Khullar V, Digesu GA (2013) Biomarkers in overactive bladder. Int Urogynecol J 24: 1065–1072. 23314226 10.1007/s00192-012-2027-1

[7] Steers WD, Tuttle JB (2006) Mechanisms of disease: the role of nerve growth factor in the pathophysiology of bladder disorders. Nat Clin Pract. Urol 3: 101–110. 10.1038/ncpuro0408 16470209

[8] Steers WD, Kolbeck S, Creedon D, Tuttle JB (1991) Nerve growth factor in the urinary bladder of the adult regulates neuronal form and function. J Clin Invest 88: 1709–1715. 1939656 10.1172/JCI115488 PMC295710

[9] Tuttle JB, Steers WD, Albo M, Nataluk E (1994) Neural input regulates tissue NGF and growth of the adult rat urinary bladder. J Auton Nerv Syst 49: 147–158. 7806767 10.1016/0165-1838(94)90134-1

[10] Vizzard MA (2001) Alterations in neuropeptide expression in lumbosacral bladder pathways following chronic cystitis. J Chem Neuroanat 21: 125–138. 11312054 10.1016/s0891-0618(00)00115-0

[11] Dupont MC, Spitsbergen JM, Kim KB, Tuttle JB, Steers WD (2001) Histological and neurotrophic changes triggered by varying models of bladder inflammation. J Urol 166: 1111–1118. 11490308

[12] von Elm E, Altman DG, Egger M, Pocock SJ, Gøtzsche PC, et al (2007) The Strengthening the Reporting of Observational Studies in Epidemiology (STROBE) statement: guidelines for reporting observational studies. Epidemiology 18: 800–804. 18049194 10.1097/EDE.0b013e3181577654

[13] Murphy GK, McAlister FA, Weir DL, Tjosvold L, Eurich DT (2014) Cardiovascular medication utilization and adherence among adults living in rural and urban areas: a systematic review and meta-analysis. BMC Public Health 14: 544. 24888355 10.1186/1471-2458-14-544 PMC4064809

[14] Higgins JP, Thompson SG (2002) Quantifying heterogeneity in a meta-analysis. Stat Med 21: 1539–1558. 12111919 10.1002/sim.1186

[15] Zintzaras E, Ioannidis JP (2005) Heterogeneity testing in meta-analysis of genome searches. Genet Epidemiol 28: 123–137. 15593093 10.1002/gepi.20048

[16] Peters JL, Sutton AJ, Jones DR, Abrams KR, Rushton L (2006) Comparison of two methods to detect publication bias in meta-analysis. JAMA 295: 676–680. 16467236 10.1001/jama.295.6.676

[17] Liu HT, Tyagi P, Chancellor MB, Kuo HC (2009) Urinary nerve growth factor level is increased in patients with interstitial cystitis/bladder pain syndrome and decreased in responders to treatment. BJU Int 104: 1476–1481. 19522864 10.1111/j.1464-410X.2009.08675.x

[18] Liu HT, Tyagi P, Chancellor MB, Kuo HC (2010) Urinary nerve growth factor but not prostaglandin E2 increases in patients with interstitial cystitis/bladder pain syndrome and detrusor overactivity. BJU Int 106: 1681–1685. 19751258 10.1111/j.1464-410X.2009.08851.x

[19] Pinto R, Lopes T, Frias B, Silva A, Silva JA, et al (2010) Trigonal injection of botulinum toxin A in patients with refractory bladder pain syndrome/interstitial cystitis. Eur Urol 58: 360–365. 20227820 10.1016/j.eururo.2010.02.031

[20] Kuo HC, Liu HT, Chancellor MB (2010) Urinary nerve growth factor is a better biomarker than detrusor Wall thickness for the assessment of overactive bladder with incontinence. Neurourol Urodyn 29: 482–487. 19367641 10.1002/nau.20741

[21] Chung SD, Liu HT, Lin H, Kuo HC (2011) Elevation of serum C-Reactive protein in patients with OAB and IC/BPS implies chronic inflammation in the urinary bladder. Neurourol Urodyn 30: 417–420. 21284020 10.1002/nau.20938

[22] Liu HT, Kuo HC (2012) Increased urine and serum nerve growth factor levels in interstitial cystitis suggest chronic inflammation is involved in the pathogenesis of disease. PLOS ONE 7: e44687. 23028581 10.1371/journal.pone.0044687 PMC3444462

[23] Tyagi P, Killinger K, Tyagi V, Nirmal J, Chancellor MB, et al (2012) Urinary Chemokines as noninvasive predictors of ulcerative interstitial cystitis. J Urol 187: 2243–2248. 22503040 10.1016/j.juro.2012.01.034 PMC3674640

[24] Corcoran AT, Yoshimura N, Tyagi V, Jacobs B, Leng W, et al (2013) Mapping the cytokine profile of painful bladder syndrome/interstitial cystitis in human bladder and urine specimens. World J Urol 31: 241–246. 22441309 10.1007/s00345-012-0852-y PMC3674577

[25] Jiang YH, Liu HT, Kuo H-C (2014) Decrease of urinary nerve growth factor but not brain-derived neurotrophic factor in patients with interstitial cystitis/bladder pain syndrome treated with hyaluronic acid. PLOS ONE 9: e91609. 24614892 10.1371/journal.pone.0091609 PMC3948883

[26] Erickson DR, Xie SX, Bhavanandan VP, Wheeler MA, Hurst RE, et al (2002) A comparison of multiple urine markers for interstitial cystitis. J Urol 167: 2461–2469. 11992058

[27] Saini R, Gonzalez RR, Te AE (2008) Chronic pelvic pain syndrome and the overactive bladder: the inflammatory link. Curr Urol Rep 9: 314–319. 18765131 10.1007/s11934-008-0054-8

[28] Ogawa T, Homma T, Igawa Y, Seki S, Ishizuka O, et al (2010) CXCR3 binding chemokine and TNFSF14 over expression in bladder urothelium of patients with ulcerative interstitial cystitis. J Urol 183: 1206–1212. 20096889 10.1016/j.juro.2009.11.007

[29] Vizzard MA (2000) Alterations in spinal cord Fos protein expression induced by bladder stimulation following cystitis. Am J Physiol Regul Integr Comp Physiol 278: R1027–R1039. 10749792 10.1152/ajpregu.2000.278.4.R1027

[30] Amaya F, Shimosato G, Nagano M, Ueda M, Hashimoto S, et al (2004) NGF and GDNF differentially regulate TRPV1 expression that contributes to development of inflammatory thermal hyperalgesia. Eur J Neurosci 20: 2303–2310. 15525272 10.1111/j.1460-9568.2004.03701.x

[31] Steers WD (2002) Pathophysiology of overactive bladder and urge urinary incontinence. Rev Urol 4: S7–18. 16986023 PMC1476015

[32] Lamb K, Gebhart GF, Bielefeldt K (2004) Increased nerve growth factor expression triggers bladder overactivity. J Pain 5: 150–156. 15106127 10.1016/j.jpain.2004.01.001

[33] Lowe EM, Anand P, Terenghi G, Williams-Chestnut RE, Sinicropi DV, et al (1997) Increased nerve growth factor levels in the urinary bladder of women with idiopathic sensory urgency and interstitial cystitis. Br J Urol 79: 572–577. 9126085 10.1046/j.1464-410x.1997.00097.x

[34] Liu HT, Kuo HC (2007) Intravesical botulinum toxin A injections plus hydrodistension can reduce nerve growth factor production and control bladder pain in interstitial cystitis. Urology 70: 463–468. 17905097 10.1016/j.urology.2007.04.038

[35] Chuang YC, Fraser MO, Yu Y, Chancellor MB, de Groat WC, et al (2001) The role of bladder afferent pathways in bladder hyperactivity induced by the intravesical administration of nerve growth factor. J Urol 165: 975–979. 11176525

[36] Okragly AJ, Niles AL, Saban R, Schmidt D, Hoffman RL, et al (1999) Elevated tryptase, nerve growth factor, neurotrophin-3 and glial cell line-derived neurotrophic factor levels in the urine of interstitial cystitis and bladder cancer patients. J Urol 161: 438–441. 9915421

[37] Rachaneni S, Arya P, Latthe P (2013) Urinary nerve growth factor: a biomarker of detrusor overactivity? A systematic review. Int Urogynecol J 24: 1603–1609. 23649686 10.1007/s00192-013-2104-0

